# Cervical, Breast, and Colorectal Cancer Screening Outcomes Among Refugees in Philadelphia, Pennsylvania

**DOI:** 10.1007/s10903-025-01685-y

**Published:** 2025-04-05

**Authors:** Colleen Payton, Nina Kvaratskhelia, Melanie Chalfin, Jessica Deffler, Marc Altshuler

**Affiliations:** 1 Moravian University, Bethlehem, PA USA; 2 Thomas Jefferson University, Philadelphia, PA USA

**Keywords:** Refugees, Cervical cancer screening, Breast cancer screening, Colorectal cancer screening

## Abstract

Cancer screening can detect cancer at an early stage and decrease cancer morbidity and mortality. Refugee populations may have had limited access to cancer screening before arrival in the United States. A cross-sectional analysis of cervical, breast, and colorectal cancer screening was conducted among refugees with primary care visits between 2018 and 2022 at a refugee health clinic in Philadelphia, Pennsylvania. Cancer screening outcomes included the number and type of screenings; the number of normal, inconclusive, and abnormal screening results; completion of follow-up tests for inconclusive and abnormal results; and the number of cancer diagnoses. Among 149 refugee women aged 21–65, 80.5% were screened for cervical cancer at least once. Among 181 cervical cancer screenings, 89.0% were normal, 3.9% were unsatisfactory, and 7.2% were abnormal. Among 38 refugee women aged 50–74, 92.1% were screened for breast cancer at least once. Among 111 breast cancer screenings, 81.1% were normal, 11.7% were incomplete, and 7.2% were abnormal. Among 107 refugees aged 50–75, 80.4% were screened for colorectal cancer at least once. Among 189 colorectal cancer screenings, 76.2% were normal, 11.1% were inconclusive, and 12.7% were abnormal. There were 0 cancer diagnoses. Longitudinal outcomes beyond the domestic medical exam are valuable to provide insight into cervical, breast, and colorectal cancer screening among refugees in the United States. This could serve as a foundation for future quality improvement interventions to increase cancer screening.

## Introduction

### Global cancer Incidence and Mortality

Cancer remains one of the leading causes of mortality globally [1]. Estimates of the incidence and mortality in 2020 include 604,127 new cases and 341,831 deaths related to cervical cancer, 2,261,419 new cases and 684,996 deaths related to breast cancer, and 1,148,515 new cases and 576,858 deaths related to colorectal cancer [1]. The incidence (18.8 versus 11.3 per 100,000) and mortality (12.4 versus 5.2 per 100,000) of cervical cancer is higher in low or medium Human Development Index (HDI) countries compared to high HDI countries [1]. Although the incidence of breast cancer is 88% higher in high HDI countries, mortality is 17% higher in low or medium HDI countries often due to late-stage presentation [1]. The incidence of colorectal cancer is four times higher in high HDI countries, but there are higher fatality rates in low or medium HDI countries [1]. Timely cancer screening is associated with improved cancer morbidity and mortality.

### Cancer Screening Among Refugees in the United States

Refugees are people forced to leave their home country due to fear of persecution for reasons of race, religion, nationality, or membership of a particular group [2]. Refugees may have limited access to cancer screening before arrival in the United States (US) due to lack of standardized screening in low- and middle-income countries [3]. Upon arrival to the US, refugees often experience lower rates of cancer screening compared to non-refugees [4–11]. The US Preventive Services Task Force (USPSTF) guidelines outline cervical, breast, and colorectal screening recommendations [12]. Special considerations should be made to tailor health education and screen women above 65 years for cervical cancer if they have never been screened [3].

Refugees living in the US may experience socio-ecological barriers to cancer screening. Personal barriers include limited knowledge about cancer screening, a lack of symptoms, time away from work and transportation needed to get to the screening, and embarrassment or fear about testing [6, 13, 14]. System barriers include lack of health insurance, lack of usual source of care, lack of interpreter services, and gender-discordant providers and interpreters [6, 14–16]. Acute health issues may take precedence over cancer screenings during the domestic medical examination (DME), a post-arrival medical screening recommended by the Centers for Disease Control and Prevention. These barriers should be addressed with cultural humility when offering and coordinating cancer screenings for refugees.

### Purpose

The purpose of this quality improvement project was to analyze the outcomes of refugees screened for cervical, breast, and colorectal cancer screening among those who had primary care visits between 2018 and 2022 at a refugee health clinic in Philadelphia, Pennsylvania. Outcomes included (1) cancer screenings (number and type of cancer screenings completed; reasons for never completing screening), (2) cancer screening results (number of normal, inconclusive, and abnormal screening results; reasons for inconclusive results; type of abnormal results), (3) completion of follow-up tests for inconclusive and abnormal results, and (4) the number of cancer diagnoses.

## Methods

### Study Design

A cross-sectional analysis of cervical, breast, and colorectal cancer screening outcomes was conducted among refugees with a primary care visit at a refugee clinic in Philadelphia, Pennsylvania between 2018 and 2022. This was a follow-up, quality improvement project from an earlier project conducted at 3 US clinics that analyzed the proportion of refugees ever screened and up-to-date on cervical, breast, and colorectal cancer screening completed in the US by country of origin and language [17].

### Setting

Refugees received the DME at a refugee clinic in Philadelphia, Pennsylvania. Within the family medicine practice, patients may receive longitudinal care after the DME. Phone interpretation services were available 100% of the time and used unless patients defer because they are fluent in English. The refugee clinic has seen over 2,000 refugees since 2007. The Institutional Review Board at Thomas Jefferson University approved this analysis under expedited review.

### Population

The population included adult refugees, asylees, Special Immigrant Visas (SIVs), and Afghan humanitarian arrivals who had a DME by 12/31/2021 and at least one primary care visit (internal medicine, family medicine, or OBGYN) between 2018 and 2022. Inclusion criteria for each cancer screening was based on age as of 12/31/2021 and comprised women aged 21 to 65 years for cervical cancer screening, women aged 50 to 74 years for breast cancer screening, and adults aged 50 to 75 years for colorectal cancer screening. Country of origin data was described.

Patients who died during the project time period were excluded. Exclusion criteria for cervical cancer screening included documentation or self-report of hysterectomy or previous history of cervical cancer. Exclusion criteria for breast cancer screening included documentation or self-report of bilateral mastectomy or previous history of breast cancer. Exclusion criteria for colorectal cancer screening included previous history of colorectal cancer.

### Data Collection

Data was collected from the electronic health records (EHR) of the refugee clinic. Data was managed within Research Electronic Data Capture (REDCap) at Thomas Jefferson University [18, 19].

### Measures

#### Cervical Cancer Screening Outcomes

Ever screened for cervical cancer included at least one screening in the US. Up-to-date cervical cancer screening was based on 2018 USPSTF guidelines [20]. Up-to-date cervical cancer screening for women aged 21 to 29 was defined as at least 1 cervical cytology in the US in the last 3 years between 2020 and 2022. Up-to-date cervical cancer screening for women aged 30 to 65 was defined as at least 1 screening (cervical cytology or hrHPV) in the US in the last 5 years between 2018 and 2022. Normal results were defined as no abnormal cervical cells. Unsatisfactory results indicated a lab sample that could not be interpreted for reasons such as the lab sample did not have enough cells, the cells were clumped together, or the cells were hidden by mucus or blood [21]. Abnormal results indicated some of the cells look different than normal cells, which could include: atypical squamous cells of undetermined significance (ASC-US); atypical glandular cells (AGC); low-grade squamous intraepithelial lesions (LSIL); atypical squamous cells, cannot exclude high-grade squamous intraepithelial lesion (ASC-H); high-grade squamous intraepithelial lesions (HSIL); adenocarcinoma in situ (AIS); cervical cancer cells (squamous cell carcinoma or adenocarcinoma) [21].

#### Breast Cancer Screening Outcomes

Ever screened for breast cancer included at least one screening in the US. Up-to-date breast cancer screening was based on 2016 USPSTF guidelines [22]. Up-to-date breast cancer screening was defined as having at least one mammography screening in the US in the last two years between 2021 and 2022. The Breast Imaging Reporting and Data System (BI-RADS) was used to interpret the results. Normal results were defined as BI-RADS category 1 (negative). Unsatisfactory results were defined as BI-RADS category 0 (need additional imaging evaluation) [23]. Abnormal results were defined as BI-RADS category 2 (benign (not cancer)), category 3 (probably benign), category 4 (suspicious abnormality), category 5 (highly suggestive of malignancy (cancer)), or category 6 (known biopsy-proven malignancy (cancer)) [23].

#### Colorectal Cancer Screening Outcomes

Ever screened for colorectal cancer included at least one screening in the US. Up-to-date colorectal cancer screening was based on 2016 USPSTF guidelines [24]. Up-to-date colorectal cancer screening was defined as having at least one colonoscopy in the US in the last ten years between 2013 and 2022 or at least 1 fecal immunochemical test (FIT) test in the US in the last year in 2022. Normal results were defined as reported normal colon on colonoscopy or negative FIT test. Inconclusive results were defined as incomplete colonoscopy due to factors such as inadequate bowel preparation or incomplete FIT test due to factors such as inadequate sample or improper mailing of test [25]. Abnormal results were defined as an adequate colonoscopy with any abnormal finding or a FIT test with a positive result [25].

### Analysis

Descriptive statistics were calculated including counts and percentages.

## Results

### Cervical Cancer Screening Outcomes

**Fig. 1:**
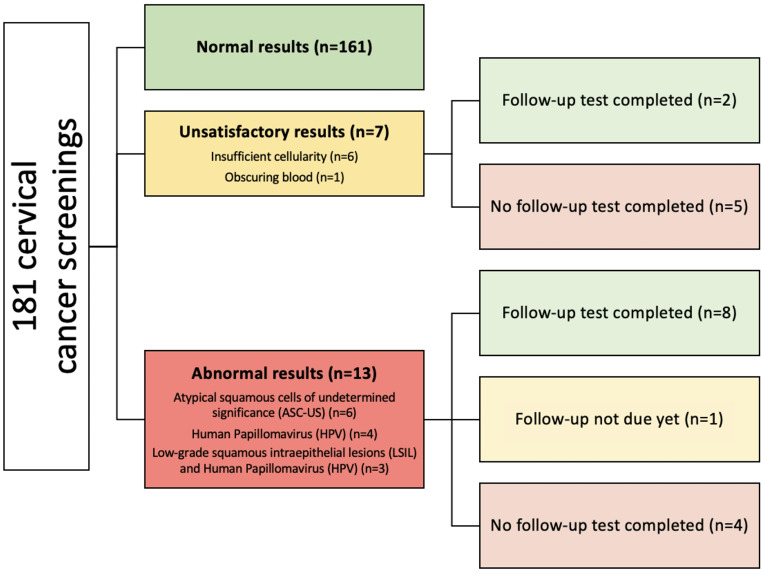
Cervical cancer screening outcomes among refugees in Philadelphia, Pennsylvania, 2018–2022 (*N* = 120)

Among 149 women, the average age was 39.78 (SD = 11.51) and the top countries of origin included Bhutan/Nepal (20.8%), Iraq (17.4%), Afghanistan (11.4%), Burma/Myanmar (10.7%), Democratic Republic of the Congo (8.7%), Syria (8.7%), and other countries with small sample sizes (22.1%). 80.5% were screened at least once, and 46.3% were up-to-date on cervical cancer screening (Table 1). There was an average of 1.51 (SD = 0.59) cervical cancer screenings per person. Among those never screened (*n* = 29), 79.3% were offered screening; reasons for not completing screening from most to least common include: limited visits, declining since not sexually active, urgent and complex health conditions taking priority at visits, wanting a female OBGYN close to home, telemedicine visits during the COVID-19 pandemic, and religious reasons. Among those never screened (*n* = 29), 15 (52%) included those aged 21–26 years old. Among 181 cervical cancer screenings, 89.0% were normal, 3.9% inconclusive, and 7.2% abnormal (Fig. 1). Reasons for inconclusive results include insufficient cellularity (*n* = 6) and obscuring blood (*n* = 1). Among 13 abnormal results, pap smears indicated ASC-US (*n* = 6), Human Papillomavirus (HPV) (*n* = 4), and low-grade squamous intraepithelial lesions (LSIL) with HPV (*n* = 3). Among the 145 cervical cancer screenings completed in women 30 years or older, 91.7% had documentation of an HPV co-test. There were 0 cervical cancer diagnoses.

**Table 1:** Prevalence of cervical, breast, and colorectal cancer screening among refugees in Philadelphia, Pennsylvania, 2018–2022

Cancer screenings	Never screened *n* (%)	Screened at least once *n* (%)	Up-to-date on screening *n* (%)
Cervical cancer screening (*N* = 149)	29 (19.5%)	120 (80.5%)	69 (46.3%)
Breast cancer screening (*N* = 38)	3 (7.9%)	35 (92.1%)	8 (21.1%)
Colorectal cancer screening (*N* = 107)	21 (19.6%)	86 (80.4%)	57 (53.3%)

### Breast Cancer Screening Outcomes

**Fig. 2:**
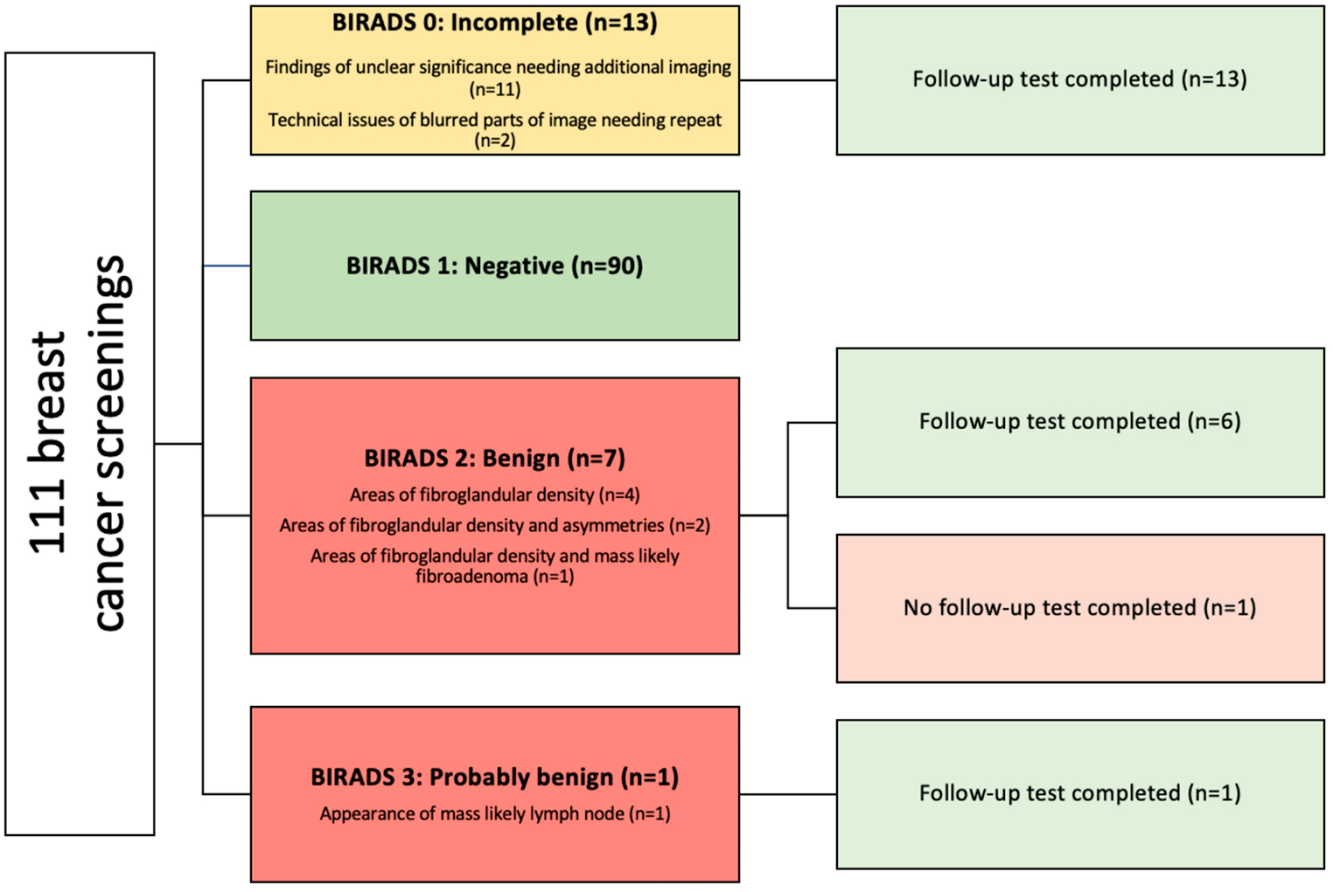
Breast cancer screening outcomes among refugees in Philadelphia, Pennsylvania, 2018–2022 (*N* = 35)

Among 38 women, the average age was 60.84 (SD = 6.11) and the top countries of origin included Bhutan/Nepal (34.2%) and other countries with small sample sizes (65.8%). 92.1% were screened at least once, and 21.1% were up-to-date on breast cancer screening. There was an average of 2.92 (SD = 2.23) breast cancer screenings per person. Among the 3 who were never screened, 2 were offered screening. Among those never screened (*n* = 3), reasons include limited clinic visits where a discussion on mammograms could occur and a patient willing to get a mammogram but not making an appointment. Among 111 breast cancer screenings, 81.1% were negative (BI-RADS 1), 11.7% incomplete (BI-RADS 0), and 7.2% abnormal (BI-RADS 2: Benign or BI-RADS 3: Probably benign) (Fig. 2). Reasons for incomplete results include findings of unclear significance needing additional imaging (*n* = 11) and technical issues of blurred parts of image needing repeat (*n* = 2). Among 8 abnormal results, mammograms indicated areas of fibroglandular density (*n* = 4), areas of fibroglandular density and asymmetries (*n* = 2), areas of fibroglandular density and mass likely fibroadenoma (*n* = 1), and appearance of mass likely lymph node (*n* = 1). There were 0 breast cancer diagnoses.

### Colorectal Cancer Screening Outcomes

**Fig. 3:**
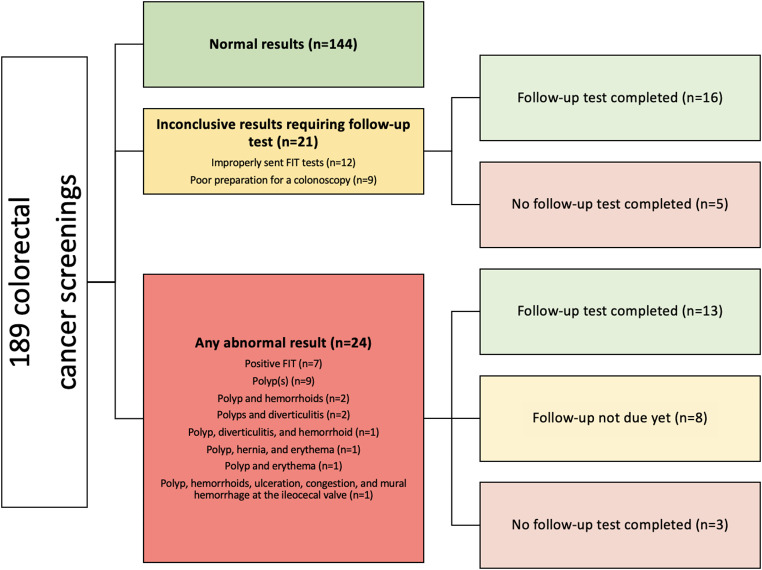
Colorectal cancer screening outcomes among refugees in Philadelphia, Pennsylvania, 2018–2022 (*N* = 86)

Among 107 adults, the average age was 61.21 (SD = 7.67) and the top countries of origin included Bhutan/Nepal (32.7%), Iraq (23.4%), and other countries with small sample sizes (43.9%). 80.4% were screened at least once, and 53.3% were up-to-date on colorectal cancer screening. There was an average of 1.77 (SD = 1.67) colorectal cancer screenings per person. Among those never screened (*n* = 21), 76.2% were offered screening; common reasons for not completing screening from most to least common include: refusing or being scared to complete a colonoscopy, willing to complete a FIT test but not completing the screening, not having a recent visit after becoming eligible for colorectal cancer screening based on age, andcomplex patients with multiple issues making it challenging to address in an office visit. Among those never screened for colorectal cancer (*n* = 21), 16 (76%) included those aged 50–55 and 70–75 years old. There were 189 colorectal cancer screenings including 129 (68.2%) FIT and 60 (31.7%) colonoscopies. Among 189 colorectal cancer screenings, 76.2% were normal, 11.1% were inconclusive results requiring follow-up, and 12.7% were abnormal (Fig. 3). Reasons for inconclusive results include poor preparation for a colonoscopy (*n* = 9) and improperly sent FIT tests (*n* = 12) such as labeling errors, not sending in the sample, and the sample sent past expiry. Among 24 abnormal results, colorectal cancer screenings indicated positive FIT (*n* = 7) or polyp(s) (*n* = 17). There were 0 colorectal cancer diagnoses.

## Discussion

### New Contribution to the Literature

This work is novel in that it examines cervical, breast, and colorectal cancer screening outcomes among refugees in the US including normal, inconclusive, and abnormal results. Compared to previous published literature among refugees living in the US, our results indicate higher rates of ever screened for cervical, breast, and colorectal cancer; our results indicate similar rates of up-to-date for cervical cancer screening, lower rates of up-to-date for breast cancer screening, and higher rates of up-to-date for colorectal cancer screening. The crude up-to-date prevalence rates for cervical (77.7% US vs. 46.3% refugees in Philadelphia), breast (78.3% in US, 78.3% in Philadelphia, and 21.1% in refugees in Philadelphia), and colorectal (74.2% in US, 63.5% in Philadelphia, and 53.3% in refugees in Philadelphia) cancer screening in the US in 2020 were higher than the rates within Philadelphia and the refugee population in Philadelphia in our project [26, 27].

Our results indicate a trend of lower rates of abnormal findings compared to the general population indicating that there were not late-stage cancer diagnoses being made, which could be due to the small sample. The abnormal results demonstrate results that could potentially require follow-up and could potentially turn into cancer. For example, polyps identified during a colonoscopy could be removed before they turn into cancer. The follow-up testing after an abnormal result demonstrates the number of refugees who were able to navigate the healthcare system to complete follow-up testing. Refugees are at an increased risk for late-stage cancer diagnoses as they may have limited access to cancer screenings before arrival in the US.

Quality improvement projects are meaningful to better understand reasons for never completing screening and follow-up of both inconclusive and abnormal results. Age is an important factor to address to initiate cervical cancer screening and to both initiate and sustain colorectal cancer screening. Our clinic utilizes the medical home model and sees patients at the DME and longitudinally over time, which allows for trust to be developed. Cancer screenings were ordered shortly after the DME, which allows more time for refugees to receive cancer screenings and related follow-up if applicable. Potential reasons for our higher cancer screening rates among refugees could be related to resources for health navigation including a combination of clinic liaisons, community health workers, and students to connect patients to care. Our clinic also has several female providers, which increases the completion of cervical cancer screening.

### Cervical Cancer Screening

Literature on cervical cancer screening among refugees living in the US varies from 13.9-73% ever screened and 41.4–90.6% up-to-date [5–8, 11]. Our results indicate higher ever screened (80.5%) and similar up-to-date screening (46.3%). There was a similar rate of ever screened in our population mainly from Bhutan/Nepal, Iraq, Afghanistan, Burma/Myanmar, Democratic Republic of the Congo, and Syria compared to refugees from Bhutan (47% and 90%), Iraq (64%), and Myanmar (43% and 55%) described in the literature [5, 7, 28]. Cervical cancer screening is important among refugees because of lower rates of HPV vaccination overseas [3]. Of the 7 patients with unsatisfactory results, 5 did not complete follow-up testing indicating a potential barrier to care. Our results indicated that 7.2% of cervical cancer screening results were abnormal including either ASCUS or LSIL, which are lower risk abnormal cytology [21]. This is lower than abnormal pap smears for the general US population, which is closer to 16.4% [29]. Of the 13 patients with abnormal results, only 4 did not complete follow-up testing. Our clinic has a strong partnership with clinical liaisons at the resettlement agency, which allows for more efficient scheduling and follow-up of abnormal cancer screenings; the clinic liaisons can escort patients to new healthcare locations during the first few months, which builds trust among the community. Additionally, our clinic has a team of physicians, nurses, community health workers, and a social worker. At our clinic, 8.3% of the cervical cancer screenings completed in women 30 years or older were for cytology alone. Our EMR defaults to every 5 years for cervical cancer screening health maintenance, but future work could tailor this timeframe for every 3 years in women who did not have an HPV co-test.

### Breast Cancer Screening

Literature on breast cancer screening among refugees living in the US varies from 33-75% ever screened and 15.8–46.4% up-to-date [6, 7, 10, 11, 30]. Our results indicate higher ever screened (92.1%), but lower up-to-date screening (21.1%). This was a higher rate of ever screened than previous literature in a similar population from Bhutan/Nepal (69%) [7]. Since most refugees have not had preventive screening before US arrival, the clinic and the resettlement agency worked closely to schedule patients for mammograms within the first few months of arrival. The up-to-date breast screening rate may have been most affected by the COVID-19 pandemic as the timeframe for completion of screening was between 2020 and 2022 [31]. Among 111 breast cancer screenings in our project, 8 (7.2%) were abnormal. About 10% of mammogram results from the literature in the general population were abnormal leading to further testing [32]. Our results indicate a high completion of follow-up for incomplete (BIRADS 0) or abnormal (BIRADS 2 and 3) results. However, previous research indicates that there are delays in follow-up of abnormal mammograms among non-English language speakers [33]. Radiologists may request follow-up diagnostic imaging for abnormal screening results, which may be difficult to coordinate among refugees beyond the initial period of resettlement. A future quality improvement project could employ a patient navigator to outreach to women who are not up-to-date on breast cancer screening by making the appointment while the patient is on the phone [34].

### Colorectal Cancer Screening

There is a particular need for more published literature on colorectal cancer screening among refugees living in the US. Colorectal cancer screening rates for refugees living in the US vary from 14-38.5% ever screened and 14–32% up-to-date [6, 7, 11]. Our results indicate a higher proportion of refugees ever screened (80.4%) and up-to-date (53.3%). This higher rate of ever screened could be related to the higher proportion of FIT tests (68.2%). There was a higher rate of ever screened than previous literature among refugees from similar countries including Iraq (14%), Bhutan (17%), and Myanmar (25%) [6, 7]. Among 189 colorectal cancer screenings in our project, 24 (12.7%) were abnormal including 7 of 129 (5.5%) FIT and 17 of 60 (28.3%) colonoscopies. Results from the literature indicate that 8.5% of FIT test results from the general population were positive [35]. Results from the literature indicate that about 40% of colonoscopies included polyps [36].

Our clinic reviews both FIT tests and colonoscopies with all eligible patients and has FIT kits readily available in the office to hand out. FIT test instructions should be available in multiple languages. Previous studies have shown that text messaging with an opt-out mailed FIT test boosts colorectal cancer screening rates, particularly among underserved populations in Philadelphia, Pennsylvania [37]. While colonoscopies remain the gold standard in colorectal cancer screenings, FIT tests have been shown to have a sensitivity of 80% in the detection of colon cancer and are significantly less time-intensive and invasive than colonoscopy [38]. The higher rate of up-to-date screening could be related to colonoscopies being completed more recently and the ten year interval in between normal colonoscopies. Some patients were nervous to complete a colonoscopy, and inconclusive results were related to poor preparation for a colonoscopy. There may be fewer barriers for newcomers to complete a FIT test with patient navigation while physician-patient rapport is being established [39, 40]. Future quality improvement projects could work to develop a colorectal cancer screening education and toolkit; healthcare providers could work to improve communication to reduce improperly sent FIT tests including clear directions on how to properly label and mail the sample within the timeframe.

### Limitations

Cancer screening results may not be generalizable to all refugees in the US. Results could not be separated out by specific country of origin due to small sample sizes. The data collection timeframe overlapped with the COVID-19 pandemic, when cancer screenings in the US significantly decreased [41, 42]. Screenings may have been underestimated if patients received screenings at other clinical sites. Up-to-date screening may overestimate those that would be up-to-date as recent arrivals who received an initial screening would be considered up to date. Although there was relatively high follow-up for abnormal results, the up-to-date screening calculations may be slightly overestimated if patients were recommended to get screened within a timeframe shorter than the standard timeframes outlined. Patients’ age was determined on 12/31/2021, which could have restricted the population eligible in 2022. Up-to-date cervical cancer screening for women aged 30 to 65 years was simplified to 5 years as most patients received cotesting, which may have slightly increased the screening rates as the guidelines state that those who received cervical cytology alone should have another screening in 3 years.

## Conclusion

Longitudinal follow-up beyond the DME is valuable to provide insight into cervical, breast, and colorectal cancer screening among refugees in the US. There is a need for quality improvement interventions to increase cancer screening and ensure follow-up. Quality improvement data may guide future interventions to address reasons for never completing screening and follow-up for inconclusive and abnormal results. Future initiatives may include assessing hesitancy and bridging barriers of sensitive exams. This may include cervical cancer screenings by female providers in patients’ communities, patient navigation for scheduling mammograms, or health education on how to collect FIT tests. This is especially prudent as refugees may not have as much support beyond the first year after resettlement, and cancer screening is important to detect cancer early and decrease cancer mortality.
